# Lower All-Cause Mortality Risk in Females and Males with Peripheral Artery Disease following Pain-Free Home-Based Exercise: A 7-Year Observational Study

**DOI:** 10.3390/jpm13040636

**Published:** 2023-04-05

**Authors:** Nicola Lamberti, Luca Traina, Caterina Savriè, Elpiniki Tsolaki, Natascia Rinaldo, Sofia Straudi, Franco Guerzoni, Nicola Napoli, Roberto Manfredini, Vincenzo Gasbarro, Fabio Manfredini

**Affiliations:** 1 Department of Neuroscience and Rehabilitation, University of Ferrara, Via L. Borsari 46, 44121 Ferrara, Italy; nicola.lamberti@unife.it (N.L.); natascia.rinaldo@unife.it (N.R.); sofia.straudi@unife.it (S.S.); fabio.manfredini@unife.it (F.M.); 2 Unit of Vascular and Endovascular Surgery, University Hospital of Ferrara, Via Aldo Moro 8, 44124 Ferrara, Italy; l.traina@ospfe.it (L.T.); niki.tsolaki@gmail.com (E.T.); vincenzo.gasbarro@unife.it (V.G.); 3 Clinica Medica Unit, University Hospital of Ferrara, Via Aldo Moro 8, 44124 Ferrara, Italy; caterina.savrie@unife.it; 4 Unit of Rehabilitation Medicine, University Hospital of Ferrara, Via Aldo Moro 8, 44124 Ferrara, Italy; 5 Health Statistics Unit, University Hospital of Ferrara, Via Aldo Moro 8, 44124 Ferrara, Italy; f.guerzoni@ospfe.it (F.G.); n.napoli@ospfe.it (N.N.); 6 University Center for Studies on Gender Medicine, Department of Medical Sciences, University of Ferrara, Via Fossato di Mortara, 64/B, 44121 Ferrara, Italy; 7 Department of Medical Sciences, University of Ferrara, Via Fossato di Mortara, 64/B, 44121 Ferrara, Italy

**Keywords:** exercise, intermittent claudication, peripheral arterial disease, peripheral vascular diseases, walking, women, sex difference

## Abstract

We evaluated the sex-specific difference in response upon participation in an exercise program with respect to the risk of adverse clinical outcomes among patients with peripheral artery disease (PAD) and claudication. The records of 400 PAD patients were assessed between 2012 and 2015. Two hundred of them were addressed to a walking program prescribed at the hospital and executed at home at symptom-free walking speed (Ex), while the remaining 200 acted as a control group (Co). The number and date of deaths, all-cause hospitalizations, and amputations for a 7-year period were collected from the regional registry. At baseline, no differences were observed (M_EX_ *n* = 138; F_EX_ *n* = 62; M_CO_ *n* = 149; F_CO_ *n* = 51). The 7-year survival rate was significantly higher in F_EX_ (90%) than in M_EX_ (82% hazard ratio, HR: 0.542 95% CI 0.331–0.885), F_CO_ (45%, HR: 0.164 95% CI 0.088–0.305), and M_CO_ (44%; HR: 0.157 95% CI 0.096–0.256). A significantly lower rate of hospitalization (*p* < 0.001) and amputations (*p* = 0.016) was observed for the Ex group compared to the Co group, without differences by sex. In conclusion, in PAD patients, active participation in a home-based pain-free exercise program was associated with a lower rate of death and better long-term clinical outcomes, particularly among women.

## 1. Introduction

Cardiovascular diseases (CVDs) are the leading cause of death globally, accounting for an estimated 17.9 million lives each year [1]. Their incidence, which is lower in premenopausal women, rises after the menopausal transition, possibly due to the loss of the cardioprotective role of sex steroid hormones and their receptors [2,3,4,5,6]. In light of the fact that biological factors may be responsible for sex-specific protective and harmful effects, sex differences may impact the presentation, development, clinical management, and outcome of CVD, including peripheral artery disease (PAD) [6,7,8,9]. This expression of systemic atherosclerosis, which affects more than 6 million individuals ≥40 years of age [6], may affect mobility and is associated with higher rates of cardiovascular death and major cardiovascular events [10,11,12].

In terms of presentation, among women, the lower limb symptoms typical of PAD may be more frequently absent, atypical, or underrecognized for concomitant musculoskeletal or osteoarticular diseases [13,14]. However, their functioning may be worse than that of men, with women showing lower functional status, lower limb strength, and faster functional decline [15,16,17,18]. In terms of outcomes, the data from invasive treatments are contradictory. Worse clinical outcomes are reported for women, such as a higher rate of adverse events, postoperative outcomes and in-hospital mortality, and discharge to a nonhome facility after surgery [19,20,21,22,23,24,25]. Conversely, equal or even better outcomes are also reported among females when compared to males [26,27,28]. However, in addition to a lower willingness to undergo vascular surgery interventions [29], women are also undertreated or poorly adherent to risk factor control therapy [30,31,32,33].

Considering all these issues, rehabilitation, which in PAD is a recommended alternative to revascularization at intermediate stages of disease [34,35] due to its capacity to counteract walking disability and cardiovascular risk [35], may represent a crucial intervention for women’s health. Data from the SWEDEHEART registry showed that participation in an exercise-based rehabilitation program was associated with reduced total mortality, particularly in women [7]. Unfortunately, for rehabilitative outcomes, sex differences are also reported, with participation in exercise programs in PAD, as in various chronic diseases, being limited by barriers in terms of referral, enrollment, and completion and outcomes, particularly among women [36,37,38]. Conversely, a structured pain-free exercise program for PAD patients prescribed at a hospital and performed at home designed to reduce most barriers to exercise [39,40] proved to obtain adherence and functional outcomes without gender differences [41,42].

In the absence of data reporting the long-term outcomes following rehabilitation in PAD, we investigated whether sex differences in clinical outcomes and rate of survival are observable at the 7-year follow-up in a cohort of patients discharged from the mentioned structured home-based program [39] compared to a cohort of PAD patients not exposed to rehabilitation.

## 2. Materials and Methods

### 2.1. Study Design and Setting

This is a single-center cohort study carried out at the University Hospital of Ferrara. The Local Ethics Committee (CE-AVEC) approved the study (number 277/2019), but written informed consent was not attainable from all participants. The study is reported according to the STROBE guidelines [43].

This single-center study retrospectively analyzed a prospectively collected database of PAD patients who were studied at the Units of Vascular Surgery or at the Unit of Clinica Medica and partly referred for the training program at the Unit of Rehabilitation Medicine.

A total of 400 consecutive PAD patients were recruited between 2012 and 2015. The follow-up period started from their recruitment and lasted for 7 years. Data concerning long-term outcomes were gathered from the Emilia-Romagna health service registry.

### 2.2. Participants

Two hundred PAD patients at Leriche-Fontaine’s stage II were enrolled in the rehabilitation program. Patients with ischemic rest pain or foot ulcers or with severe cardiovascular conditions contraindicating exercise were excluded.

A parallel group of another 200 PAD patients with the same inclusion and exclusion criteria was also studied from the cohort of patients referred to the Units of Vascular Surgery and Clinica Medica in the same temporal frame. These patients were not referred to the rehabilitation program, or they refused or postponed participation.

According to study objectives, the patients were then categorized into four subgroups: males and females who underwent the exercise program (M_EX_ and F_EX_) and control males and females (M_CO_ and F_CO_).

### 2.3. Exercise Group

All patients received the “test in–train out” home-based exercise program [39,40]. The structured exercise was prescribed at the hospital during circa-monthly visits and was executed at home. The program encompassed two daily 10-min sessions of intermittent walking (with a 1:1 walk:rest ratio) at a prescribed speed. The training speed, converted into a walking cadence (steps/minute) and paced at home by a metronome, was slower than the individual’s walking speed at the beginning and increased weekly. The walking speed was increased by approximately by 3±1 steps per minute each week. A record of the training sessions was requested and collected at each visit. The patients were discharged from the program when a satisfactory improvement in the pain-free walking distance was attained, usually within 9 months. More details on the exercise program are reported elsewhere [39,40].

### 2.4. Control Group

Patients in the control group were treated with optimal medical therapy and advised to maintain a healthy and active lifestyle, according to the guidelines and follow-up visits from the vascular surgeons [35].

### 2.5. Study Variables

The primary outcome was survival probability, assessed from the date of enrollment to the following seven years. For patients who did not reach that follow-up limit, data were censored on 30 June 2020.

Secondary outcomes included all-cause hospitalizations and amputations. In this secondary analysis, both minor and major amputations were considered; in case of multiple procedures, only the first one was considered. Additionally, for the secondary outcomes, in patients who did not reach the 7-year follow-up limit, data were censored at the date of death, if present, or at the date of 30 June 2020.

### 2.6. Statistical Analysis

A Shapiro-Wilk test was performed to verify the data distribution. Differences in baseline characteristics for the two groups and the four subgroups were assessed using Chi-squared tests, Student’s *t* test or Mann-Whitney test, or one-way analysis of variance or Kruskal-Wallis test, as appropriate. Since no baseline differences were present in the four subgroups, a propensity-score matched analysis was not performed.

Kaplan–Meier estimates of the distribution of time from enrollment to date of the event (death, hospitalization, amputation) and a log-rank test for trend were used to compare the curves of the patient subgroups.

Multivariate Cox proportional hazards regression analyses were used to analyze the effect of several predictor variables on the primary outcome for each subgroup. Because of the limited number of events, multivariate hazard ratios (HRs) were calculated using a forward approach, with an entry limit of *p* < 0.05. A *p* value < 0.05 was considered statistically significant.

All statistical analyses were performed using MedCalc Statistical Software version 20.216 (MedCalc Software bvba, Ostend, Belgium).

## 3. Results

The clinical records of 400 patients were successfully assessed.

The final sample for the study included 113 females (51 belonging to the F_CO_ subgroup and 62 to the F_EX_ subgroup) and 287 males (149 M_CO_ and 138 M_EX_). At baseline, the four subgroups under study did not differ in any demographic or clinical characteristics (Table 1) or in PAD severity.

### 3.1. Exercise Group

All patients included in the Exercise group completed the exercise program that lasted a median of 253 (interquartile range 187–322) days, with a median of 6 (interquartile range 4–9) serial visits.

Adherence to the exercise program was high, with a mean value of 85% of the prescribed exercise sessions completed. No differences in compliance with rehabilitation were observed between males and females (85 vs. 86%, respectively; *p* = 0.80).

No adverse events occurred during the testing procedures, and none were reported during the home walking sessions.

### 3.2. Survival Probability

The median follow-up time was 6.25 years (interquartile range 4.8–7.0), and there were 143 deaths during the follow-up period, 34 in females (30%) and 109 in males (38%). Thirty-one patients died in the Exercise group, and 112 died in the Control group. Finally, according to the subgroup division, the survival rate was 90% in F_EX_, 82% in M_EX_, 45% in F_CO,_ and 44% in M_CO_. The number of deaths that occurred in each subgroup is reported in Table 2.

**Figure 1:**
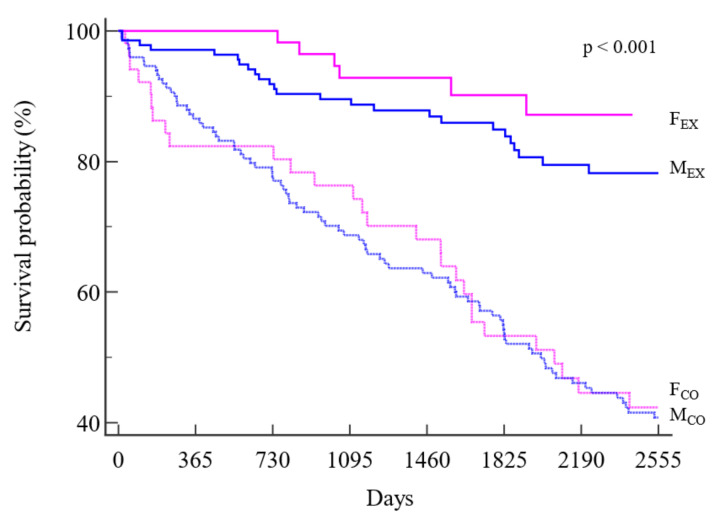
Kaplan-Meier survival curves of survival in the four subgroups. Legend: dotted lines: control group; straight lines: exercise group; pink: females; blue: males.

Kaplan–Meier analyses (Figure 1) showed a significantly lower mortality risk for the F_EX_ subgroup than for all the other subgroups, including M_EX_. In addition, a significant difference was observed for M_EX_ with respect to control patients of both sexes. The hazard ratios and the corresponding 95% confidence intervals are reported in Table 3.

### 3.3. Predictors of Survival Probability

Age (HR: 1.09; 1.06 to 1.11), chronic kidney disease (HR: 2.04; 1.45 to 2.87), and control group (HR: 4.40; 2.93 to 6.59) were the only predictors of mortality in the entire population, according to multivariate Cox regression. No differences were observed according to sex, although an almost-significant protective factor was calculated for females (HR: 0.68; 0.46 to 1.01).

The multivariate analyses conducted in the four subgroups confirmed the results of the entire population (Table 4).

### 3.4. Secondary Outcomes

A total of 312 patients underwent hospitalization, divided into 66% in F_EX_, 68% in M_EX_, 88% in F_CO,_ and 89% in M_CO_, with a significantly lower hospitalization risk (log-rank *p* < 0.001) for F_EX_ (HR: 0.62; 95% CI 0.45 to 0.87) and M_EX_ (HR: 0.62; 95% CI 0.47 to 0.80) than for M_CO_.

**Figure 2:**
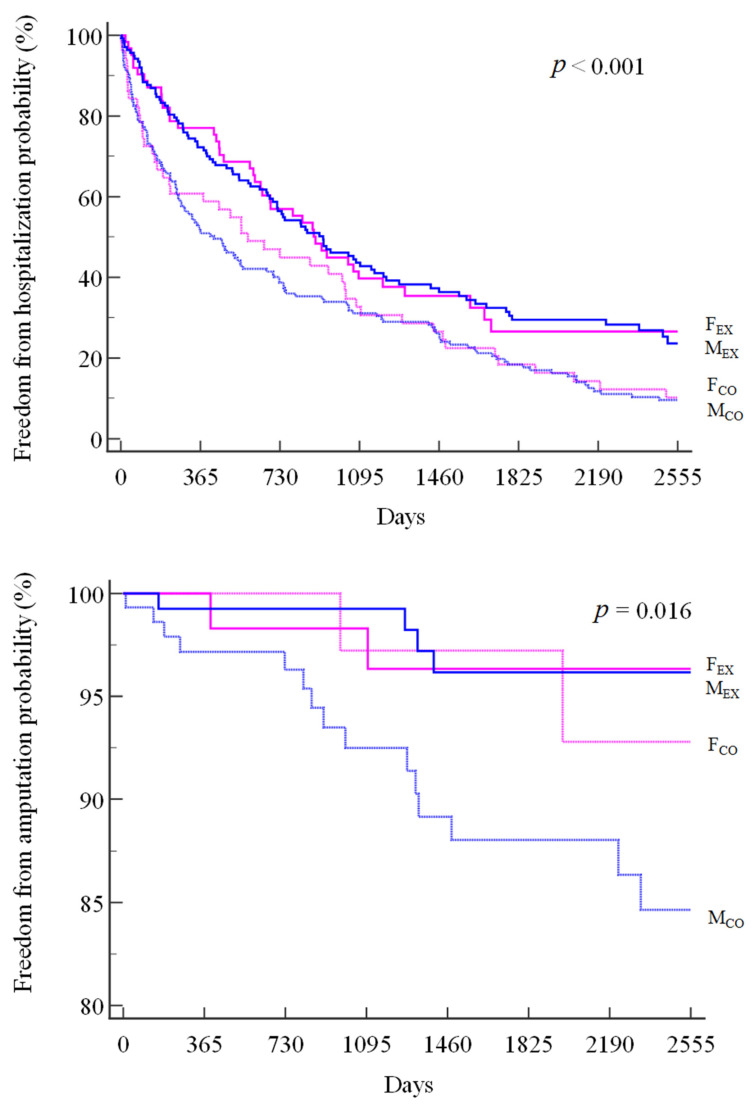
Kaplan-Meier curves of all-cause hospitalizations (top panel) and amputations (bottom panel) in the four subgroups.

A similar pattern was observed for amputations, which occurred in 3% of patients in the F_EX_ and M_EX_ groups, 4% in the F_CO_ group, and 10% in the M_CO_ group (log-rank *p* = 0.016), with a significant between-group difference for F_EX_ (HR: 0.26; 95% CI 0.08 to 0.90) and M_EX_ (HR: 0.24; 95% CI 0.09 to 0.64) when compared to M_CO_. (Figure 2)

## 4. Discussion

The study showed that in a PAD population, participation, and adherence to a home rehabilitation program were associated with a lower rate of mortality at the 7-year follow-up compared to patients receiving usual care, with active women deriving greater benefit than men. In addition, better long-term rates of hospitalizations and amputations were observed following the rehabilitation program, in this case without sex differences, compared to subjects of both sexes not undergoing rehabilitation.

The study emphasizes the impact of rehabilitation on a disease such as PAD, which is responsible for restricted mobility and low functioning, particularly in women [10,13,15,16], with associated high cardiovascular risk [7,10,11]. Among PAD patients in general, a high mortality rate ranging from 15% to 40–45% is reported in studies with a follow-up at 1 to 7 years [11,13,44], with greater impact among symptomatic patients or at higher severity of peripheral disease [7,11,13]. In addition, a high rate of nonfatal cardiovascular events has been reported [45], ranging from 15% annually to 21% in 5 years [11,44].

From this perspective, at the intermediate stages of the disease, the recovery of mobility is a crucial preventive issue. Revascularization is a possible option, but with negative and even definitely nonunivocal reports of unfavorable sex- and gender-related gaps [44,46,47].

In particular, in terms of preference, a negative attitude of women to undergoing stenting or atherectomy was reported [29], as well as to undergoing surgery for abdominal aortic aneurysm and for PAD compared with males with the same disease [22].

In terms of outcomes, vascular procedures were less successful in women [48]. In particular, following endovascular treatment, women showed higher rates of death or major amputation, higher rates of major adverse lower-limb events, and procedural complications than men [24], and higher rates of occlusion and reintervention [29]. Moreover, early graft failure and higher length of stay among black females compared to white men following lower extremity bypass [49] were reported, as well as poor outcomes among young females following iliac artery stenting [50], worse postoperative outcomes after lower extremity bypass surgery, and loss of independence [19]. Finally, similar procedural success but a higher rate of vascular complications, transfusions, and embolism [51] as well as a higher risk of wound complications [52] were also noted in women.

Considering these issues, in the intermediate stages of the disease, rehabilitative exercise may exert a preminent role [35,53] considering the unfavorable cost-effectiveness of revascularization [13,54,55], the risk of a second intervention after the first revascularization, and poor outcomes [44,46,47]. However, exercise therapy also has a complementary role after revascularization [46,56].

The present study supports the importance of rehabilitation in PAD in terms of protection. If the 7-year mortality outcome observed for the usual care group was almost within the range reported in the literature [13], the outcome in the active cohort appears dramatically more favorable. The patients with PAD after performing a structured home-based protocol at 7 years showed a double rate of survival when compared to controls, with a 94% survival rate in women. This remarkable outcome among women also confirms the data among patients after myocardial infarction, with a lower 5-year risk of mortality after rehabilitation, which is particularly favorable for women [7].

If the predictive importance of exercise capacity on the long-term mortality of PAD patients is known [57], the long-term effects of participation in exercise programs on PAD are poorly reported. In a previous study in a population of more than 1000 PAD patients undergoing the rehabilitative program reported here, we observed a worse 10-year outcome for those who discontinued the program for non-health reasons [58]. Furthermore, we observed that among patients who completed the program, the most favorable clinical outcomes were observed for those persons who gained at least 0.4 km/h or 0.1 m/s of maximal speed during rehabilitation in an incremental test to exhaustion, independent of the baseline value [59]. This change in exercise capacity was related to an increase in walking speed, a protective factor according to the literature [60,61], as well to an improvement in cardiovascular fitness as calculated peak oxygen consumption, which increased by approximately 6–10% with respect to the individual baseline level. Additional preventive actions are represented by the educational activity on risk factor management developed during the on-site visits by the progressive reintroduction of a more active lifestyle at discharge from rehabilitation and in subjects with limitations [62,63], plus a rapid improvement of night-time leg symptoms with recovery of sleep quality [64].

A further positive outcome is represented by the lower number of hospitalizations for all causes observed in the exercise group compared to the controls, in this case without a difference between sexes. It is interesting to note that the difference between the active and nonactive groups is already evident at 6 months from the start of the observation. A similar early impact was observed in dialysis patients who completed the same 6-month walking program in a multicenter trial [65].

Unfortunately, it is known that the possible beneficial effects of rehabilitation may be counteracted by barriers to participation. In the different phases of cardiac rehabilitation, which include referral, enrollment, adherence, and effect, some issues may be unsatisfactory, particularly among women compared to men [7]. Interestingly, in this study, males and females equally adhered to and completed the program, suggesting that the type and characteristics of the exercise may have positively addressed some of these barriers in favor of women, such as the in-home execution for a few minutes during the day. It is known that in addition to lack of interest, which may similarly affect both sexes in adherence to cardiac rehabilitation [8], transportation problems due to distance from the center are a strong predictor of nonattendance, as well as family obligations, which may affect women’s enrollment and program completion [8,13,66]. In addition, performance fatigability and related mechanisms, which may differ between men and women, represent a barrier, particularly for women, considering their lower physical and psychological status, comorbidities, osteoarticular disorders, and lower pain tolerance [37,67,68,69,70,71,72]. This issue may be particularly relevant in PAD, where supervised and most home-based programs are performed at a medium-to-severe degree of pain [70,73,74,75]. Conversely, pain-free programs, such as the program discussed here, based on low-intensity graded exercise, may encourage participation [37,42].

Finally, in terms of effects, different training responses may be observed between sexes, as observed in cardiac rehabilitation, possibly related to physiological determinants such as metabolic or cardiovascular factors, age, comorbidities, or a lower capacity for exercise at comparable disease severity [76,77]. In a randomized study including claudication patients comparing supervised exercise therapy (SET) under a physiotherapist’s control to walking advice, women showed a lower benefit during the first 3 months of SET and a lower walking performance after 1 year of follow-up, even in the presence of similar clinical and functional data at baseline [37]. A similar lower response among women was observed in a large cohort of patients with intermittent claudication after SET [36]. Diabetic women with PAD and claudication also showed a poor response to exercise rehabilitation compared to nondiabetic women [38].

Considering home-based programs, no significantly different response to the exercise intervention according to sex was reported [74]. At discharge from the present graded program, no different functional outcomes were observed between sexes, nor a different response to training in a fitness fatigue model of performance developed in PAD [41,42].

The limitations of the study are related to its retrospective design, with partial data in the control cohort that were impossible to retrieve. For example, patients in the control group may have participated in rehabilitation programs or community-based activities in gym clubs or manifested low adherence to risk factor control. Moreover, being an observational study, a cause-effect relationship between intervention and outcomes cannot be claimed, considering all the potential factors that may affect the results.

## 5. Conclusions

The necessity to improve cardiovascular protection and delay physical decline in women, particularly in elderly women, makes rehabilitation programs a strategic intervention. However, if tailored strategies need to be developed to improve cardiac rehabilitation participation rates equally for both sexes, this is particularly true for PAD. The favorable clinical outcomes in PAD patients, particularly among women, associated with a personalized low-intensity home-based walking program need to be confirmed in prospective randomized studies.

## Figures and Tables

**Table 1:** Baseline comparison between the four study groups.

	Males Exercise (*n* = 138)	Female Exercise (*n* = 62)	Males Control (*n* = 149)	Females Control (*n* = 51)	*p*
Age, years	72 ± 11	73 ± 11	71 ± 11	74 ± 10	0.19
Risk factors; n (%)					
Smoking	127 (92)	50 (81)	132 (89)	43 (84)	0.12
Hypertension	106 (77)	47 (76)	122 (82)	41 (80)	0.66
Hyperlipidemia	85 (62)	41 (66)	105 (70)	32 (63)	0.43
Diabetes mellitus	68 (49)	35 (57)	71 (48)	30 (59)	0.42
Chronic Kidney Disease	56 (41)	22 (36)	47 (32)	14 (28)	0.28
Comorbidities; *n* (%)					
Ischemic heart disease	69 (50)	31 (50)	76 (51)	25 (49)	0.99
Stroke	25 (18)	10 (16)	29 (20)	12 (23)	0.77
Pulmonary disease	14 (10)	4 (7)	22 (15)	5 (10)	0.38
Neoplastic disease	15 (11)	10 (16)	25 (17)	8 (16)	0.52
Age-adjusted Charlson Index	7 ± 2	7 ± 2	7 ± 2	7 ± 2	0.87
Laboratory values					
Hemoglobin, g/dL	13.8 ± 1.5	12.9 ± 1.3	13.6 ± 1.9	13.0 ± 1.4	0.18
Total Cholesterol, mg/dL	204 ± 63	209 ± 45	187 ± 45	187 ± 51	0.08
Triglycerides, mg/dL	162 ± 87	163 ± 95	135 ± 71	148 ± 59	0.14
Serum creatinine, mg/dL	1.47 ± 0.56	1.42 ± 1.02	1.57 ± 1.05	1.50 ± 1.61	0.85
Peripheral artery disease					
Lower limbs revascularization	27 (20)	7 (11)	34 (23)	7 (14)	0.18
Bilateral disease	107 (78)	53 (86)	109 (81)	41 (95)	0.06
ABI more impaired limb	0.62 ± 0.16	0.62 ± 0.17	0.61 ± 0.17	0.62 ± 0.20	0.91
ABI less impaired limb	0.83 ± 0.20	0.84 ± 0.18	0.78 ± 0.19	0.83 ± 0.22	0.19

**Table 2:** Number of deaths for each group and subgroup and hazard ratios for risk of mortality comparison between the exercise vs. control group and females vs. males.

	Deceased n (%)	Survived n (%)	Hazard Ratio (95% Conficence Interval)
Exercise Group	31 (16%)	169 (85%)	0.292 0.210 to 0.405
Control Group	112 (56%)	88 (44%)	3.423 2.470 to 4.763
Females	34 (30%)	79 (70%)	0.796 0.553 to 1.145
Males	109 (38%)	178 (62%)	1.257 0.874 to 1.808
Females Exercise	6 (10%)	56 (90%)	See Table 3.
Males Exercise	25 (18%)	113 (82%)	
Females Control	28 (55%)	23 (45%)	
Males Control	84 (56%)	65 (44%)	

**Table 3:** Hazard ratios with 95% confidence intervals for survival probability in the four subgroups.

	Females Control	Males Control	Females Exercise	Males Exercise
Females Control	-	1.049 (0.610–1.804)	0.164 (0.088–0.305)	0.303 (0.177–0.521)
Males Control	0.954 (0.554–1.640)	-	0.1566 (0.096–0.256)	0.289 (0.196–0.426)
Females Exercise	6.088 (3.274–11.321)	6.385 (3.900–10.454)	-	1.8468 (1.130–3.017)
Males Exercise	3.297 (1.921–5.658)	3.457 (2.347–5.094)	0.542 (0.331–0.885)	-

**Table 4:** Multivariate hazard ratios (95% confidence interval) of the study variables for the prediction of 7-year survival probability in the four patient subgroups.

	Males Exercise (*n* = 138)	Females Exercise (*n* = 62)	Males Control (*n* = 149)	Females Control (*n* = 51)
Age	1.11 (1.06–1.18)		1.07 (1.05–1.10)	1.07 (1.02–1.13)
Smoking				
Hypertension				
Hyperlipidemia				
Diabetes				
Chronic Kidney Disease	2.69 (1.11–6.51)	21.27 (2.12–213.62)		
Ischemic heart disease				
Stroke				
Pulmonary disease				
Neoplastic disease				4.28 (1.43–12.8)
Age-adjusted Charlson Index			1.13 (1.01–1.27)	
Lower limbs revascularization				
Bilateral disease				
ABI more impaired limb				
ABI less impaired limb				

## Data Availability

The dataset analyzed in this article is available upon reasonable request to Dr. Nicola Lamberti (nicola.lamberti@unife.it).
